# Supplementary material for: Changes in the Ultrastructure of Staphylococcus aureus Cells Make It Possible to Identify and Analyze the Injuring Effects of Ciprofloxacin, Polycationic Amphiphile and Their Hybrid
Source: Microorganisms. 2023 Aug 30;11(9):2192. doi: 10.3390/microorganisms11092192 (PMC10537381; doi:10.3390/microorganisms11092192)
Supplement: Supplementary file 1 [file microorganisms-11-02192-s001.zip › microorganisms-2504258-supplementary.pdf]

Supplementary materials

For article “Changes in the Ultrastructure of Staphylococcus aureus Cells Make It Possible to Identify and Analyze the Injuring Effects of Ciprofloxacin, Polycationic Amphiphile and their Hybrid”, by Grigor’eva, A. et al.

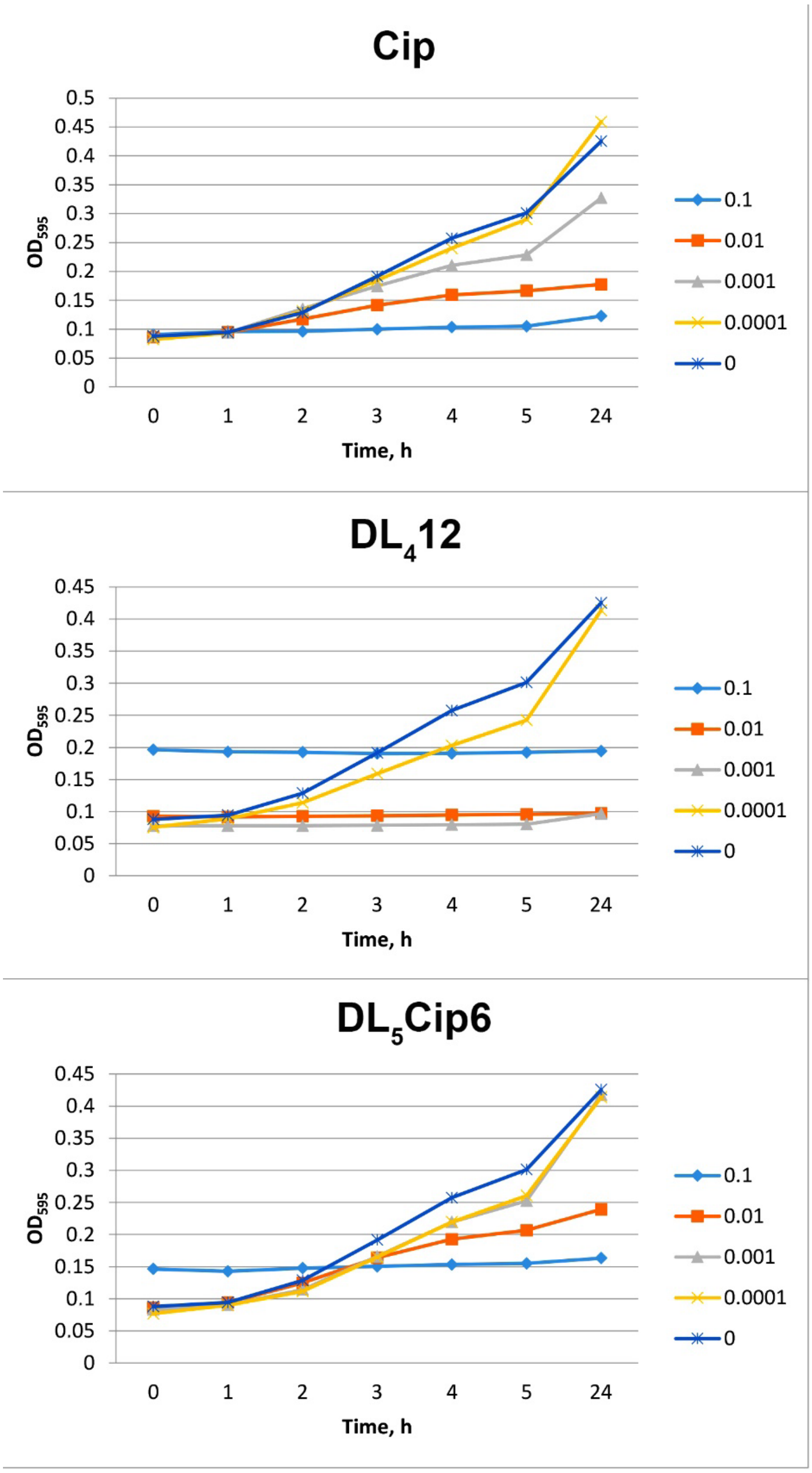

Figure S1. Growth curves of *S. aureus* in the presence of antibacterial compounds in different concentrations (μM/ml). $DL_{4}12$ and $DL_{5}Cip6$ precipitated at a concentration higher than of 0.1 mM, and therefore the OD at the zero point for this concentration differed from other.

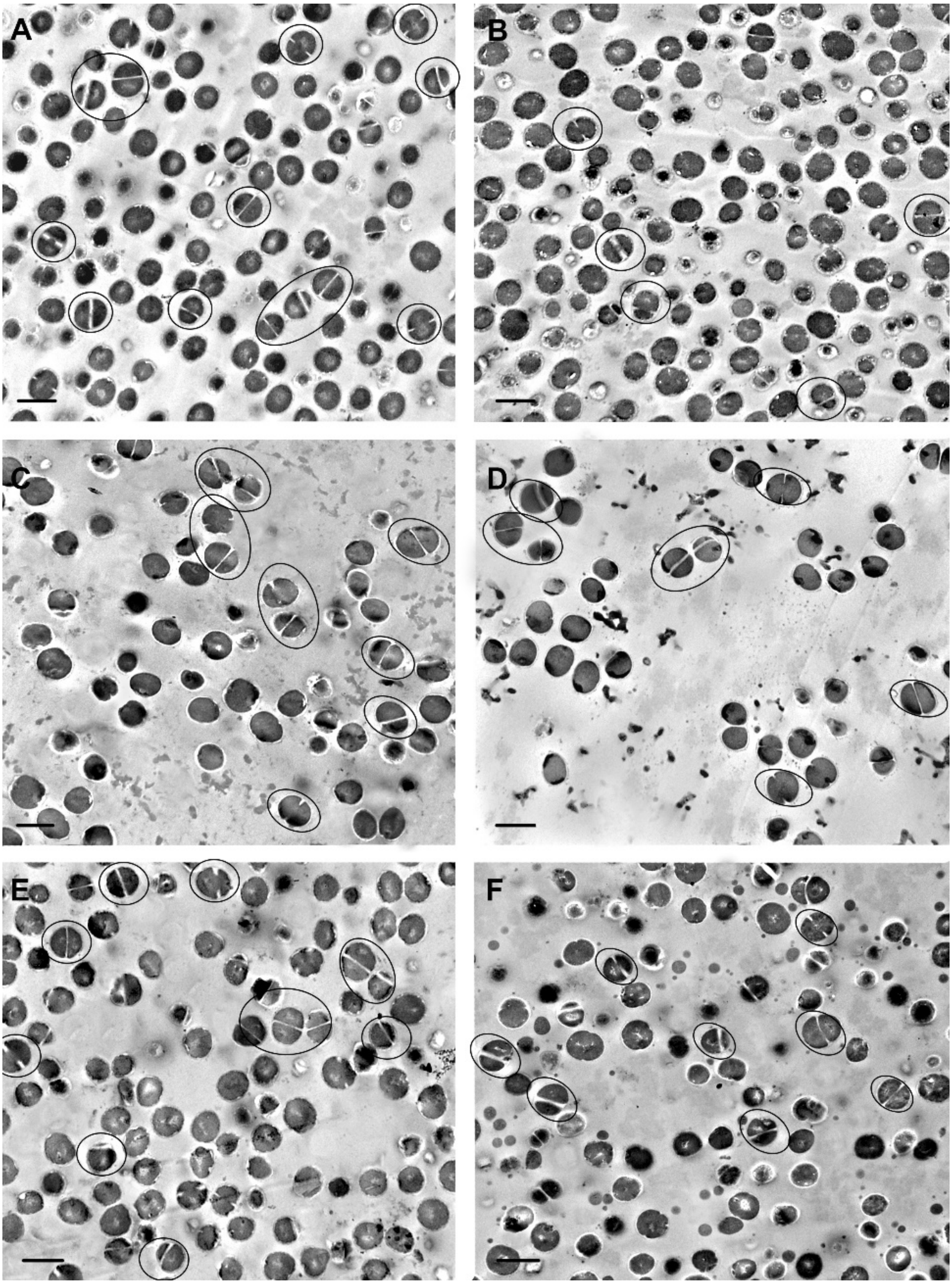

Figure S2. General view of *S. aureus* suspensions. **(A)** – intact culture; **(B)** – incubation for 45 min with Cip; **(C) -** incubation with 0.01 mM of DL$_4$12; **(D)** – with 0.1 mM of DL$_4$12; **(E)** – with 0.01 mM of DL$_5$Cip6 and **(F)** - 0.1 mM (F). **C – F** – incubation for 45 min with the preparations. The ovals show dividing cells. The length of the scale bars corresponds to 1 μm. TEM, ultrathin sections

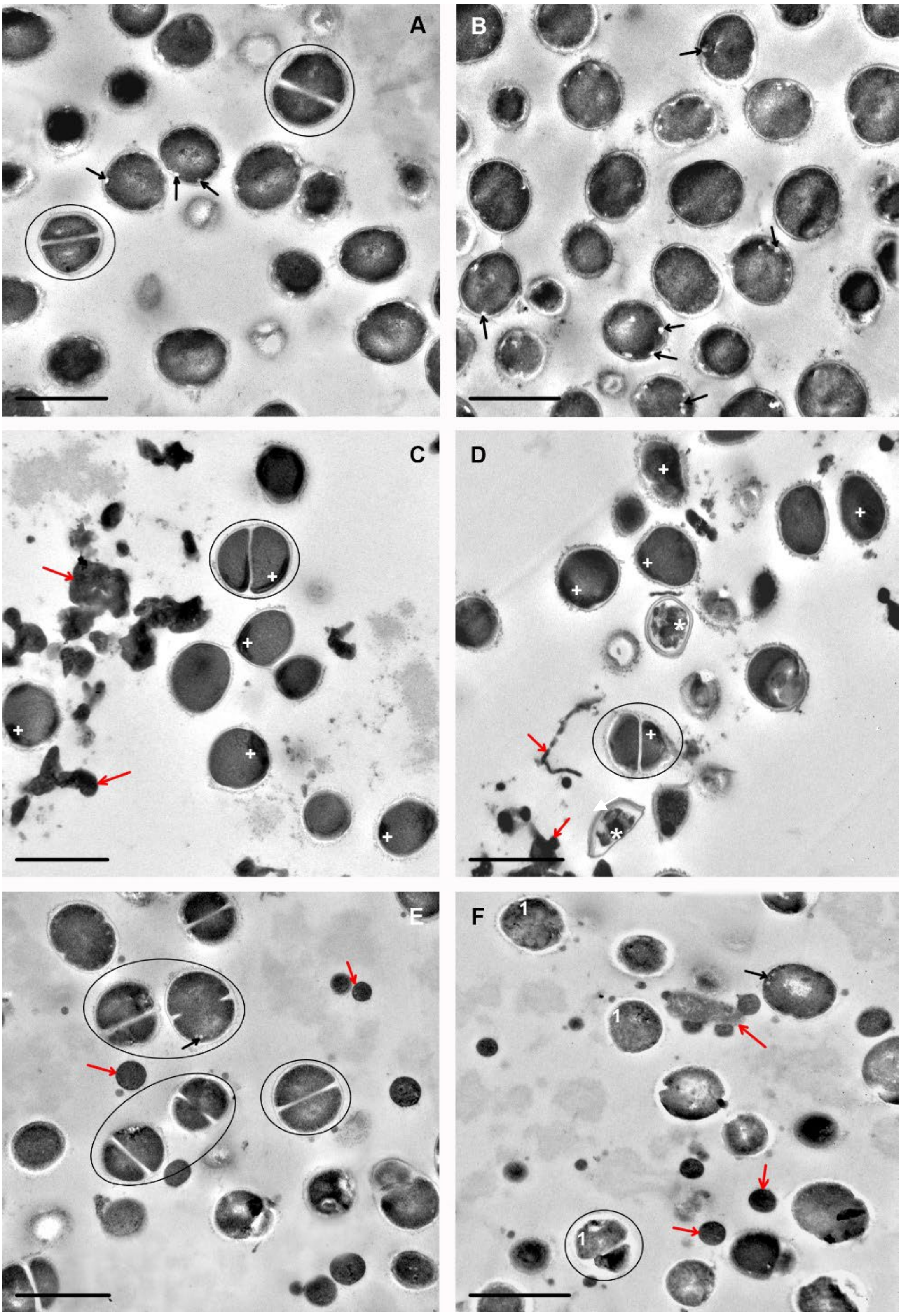

Figure S3. The ultrastructural characteristics of *S. aureus* cells incubated with antibacterial compounds: **(A, B)** – ciprofloxacin (15 and 45 min; 0.01 mM); **(C, D)** - DL$_4$12 (15 and 45 min; 0.1 mM); **(E, F)** – DL$_5$Cip6 (15 and 45 min; 0.1 mM). The ovals show dividing cells; asterisks - destroyed cells; **1** - cells with clumpy cytoplasm; white cross - accumulations of electron dense material; black arrows - spherical structures in cytoplasm; red arrows - detritus accumulation. Scale bars correspond to 1 μm. Electron microscopy, ultrathin sections.

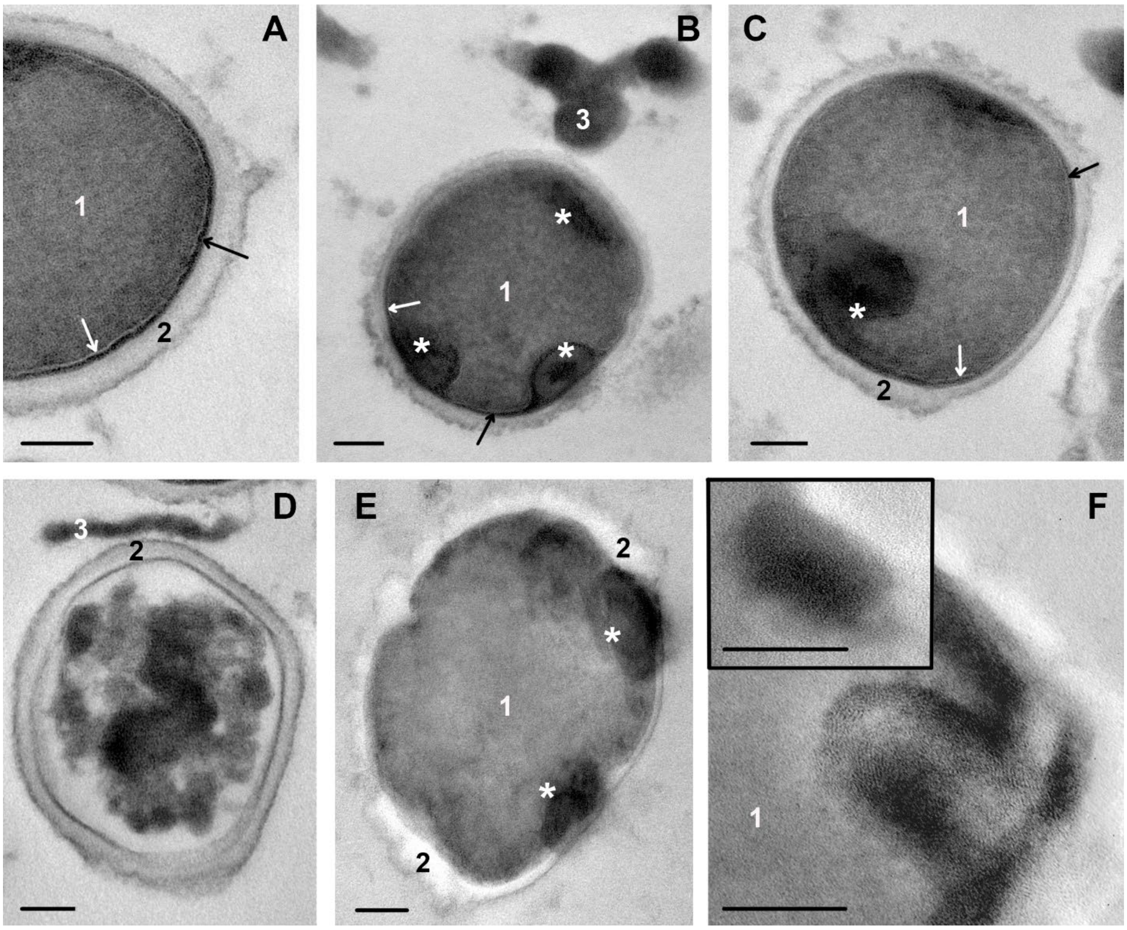

Figure S4. Ultrastructural changes in *S. aureus* cells under the influence of DL412 preparation. **(A-D)** – incubation for 15 min (0.01 mM), **(E-F)** – incubation for 45 min (0.01 mM). **(F)** – filamentous structures in electron-dense material in contrasted section, in frame: the same material with amorphous appearance in non-contrasted section. **1** – cytoplasm, **2** – cell wall; **3** – cell debris; white arrows indicate the plasma membrane, black arrows indicate the intermediate layer; asterisks - electron-dense material. Black frame borders non-contrasted section. The length of the scale bars corresponds to 100 nm.
